# Association of Genetic Variants in the Adiponectin Gene with Metabolic Syndrome: A Case-Control Study and a Systematic Meta-Analysis in the Chinese Population

**DOI:** 10.1371/journal.pone.0058412

**Published:** 2013-04-04

**Authors:** Meng Gao, Daxia Ding, Jinghua Huang, Yali Qu, Yu Wang, Qingyang Huang

**Affiliations:** 1 Hubei Key Lab of Genetic Regulation and Integrative Biology, College of Life Sciences, Central China Normal University, Wuhan, China; 2 Wuhan Center of Medical Therapeutics, Wuhan, China; 3 Department of Pharmacology and Pharmacy, the University of Hong Kong, Hong Kong, China; University of Hong Kong, China

## Abstract

**Background:**

The prevalence of metabolic syndrome has been rising worldwide, including in China, but knowledge on specific genetic determinants of metabolic syndrome is very limited. A number of studies have reported that polymorphisms in the *ADIPOQ* gene are associated with metabolic syndrome in Chinese Han populations. However, data is still conflicting. The objective of this study was to examine the associations of the adiponectin genetic variants with metabolic syndrome by a case-control study and meta-analyses in Chinese.

**Methods:**

We first investigated the association of *ADIPOQ* rs2241766 (+45T>G in exon 2), rs266729 (−11377C>G in promoter) and rs1501299 (+276G>T in intron 2) polymorphisms with metabolic syndrome in a Hubei Han Chinese population with 322 metabolic syndrome patients and 161 normal controls recruited from the Yichang, Hubei. Then we comprehensively reviewed the association between *ADIPOQ* rs2241766/rs266729/rs1501299 and metabolic syndrome in the Chinese populations via a meta-analysis. The strength of association was assessed by odds ratios (ORs) with 95% confidence intervals (CI).

**Results:**

The G allele frequency of rs2241766 in metabolic syndrome patients was significantly higher than those of controls group (29.8% vs 23.3%, OR = 1.40, *P* = 0.033). The logistic regression analysis adjusted by gender and age showed a nominally significant association for rs2241766 GG+GT genotype (*P* = 0.065, OR = 1.55) and rs1501299 GG genotype in recessive model (OR = 1.54, *P* = 0.066). However, no association was observed for rs266729 in our sample. We identified thirteen studies for rs2241766 (2,684 metabolic syndrome patients and 2,864 controls), three studies for rs266729, and eleven studies for rs1501299 (2,889 metabolic syndrome patients and 3,304 controls) in Chinese. Meta-analysis indicated significant associations for the rs2241766 G allele (OR = 1.14, 95%CI = 1.05–1.24, *P* = 0.003), rs266729 GG+GT genotypes (OR = 0.80, 95%CI = 0.68–0.92, *P* = 0.003) and rs1501299 GG+TG genotypes (OR = 1.42, 95%CI 1.16–1.75, *P* = 0.001).

**Conclusions:**

Our results demonstrated *ADIPOQ* as a pleiotropic locus for metabolic syndrome and its components in the Han Chinese population.

## Introduction

Metabolic syndrome (MetS) refers to a cluster of multiple metabolic abnormalities,including abdominal obesity, dyslipidemia (low blood levels of HDL – C, high blood levels of LDL – C and triglycerides (TG)), hypertension, insulin resistant (IR), impaired glucose tolerance (IGT), and elevated fasting glucose [1]. MetS had 4–5 fold increased risk of diabetes and 2–3 fold increased risk of heart disease and death [2]–[3]. Although there are a few versions of disputed MetS definition including the National Cholesterol Education Program Adult Treatment Panel III (ATP III) [4], the International Diabetes Federation (IDF) [5] and the World Health Organization (WHO) [6], and Chinese Diabetes Society (CDS) [7], they all agree on four major disorders, including obesity especially central obesity, IGT such as type 2 diabetes mellitus (T2DM), dyslipidemia and hypertension [1]. The prevalence of the MetS in the old population of China has reported to be 23% in men and 41% in women [8], about 21% in Chinese adults [9], 23.8% in US Whites, 21.6% in African Americans, and 31.9% in Mexican Americans [10]–[11]. The increasing prevalence of MetS poses a serious public health problem worldwide.

The familial nature of MetS, the marked difference in the prevalence among various racial groups, and differences in concordance rates between monozygotic twins clearly suggested that MetS is under genetic control. Heritability estimates for MetS range from 10% to 42% [12]–[16]. For instance, the heritability of MetS was found to be 24% among 803 individuals from 89 Caribbean-Hispanic families in the Northern Manhattan Family Study [15], 42% in 1,617 adult female twin pairs recruited from rural China with low MetS prevalence (4.4%) [16].

The evidence of genetic determinants fueled the study to identify susceptibility genes for MetS using linkage or association studies. Genome-wide linkage studies in multiple populations found evidence for linkage of MetS on chromosome 1, 2, 3q37, 7q, 16 [17]–[21]. Principal component factor analysis was also used to define quantitative phenotypes to identify the underlying genetic basis of MetS [22]–[30]. Although a number of quantitative trait loci (QTLs) were successfully identified, no specific gene or mutation has been found as a result of these linkage studies. Three genome-wide association studies (GWAS) have been carried out for MetS. Four SNPs in genes *CETP* and *LPL* are associated with MetS in Indian Asian male population [31]. GWAS of seven studies of the STAMPEED consortium, comprising 22,161 participants of European ancestry, suggested that eleven variants were nominally associated with MetS [32]. *APOA1/C3/A4/A5* gene cluster region (SNP rs964184) was associated with MetS in recent GWAS of 4 Finnish cohorts consisting of 2637 MetS cases and 7927 controls (both free of diabetes) (*P* = 7.23×10^−9^ in meta-analysis) [33].

Adiponectin (*ADIPOQ*) gene is located on human 3q27, a susceptibility locus for MetS and its components. *ADIPOQ* expresses adipocyte-specific secretory protein. The data from human studies suggested that plasma ADIPOQ level is related to each component of MetS. It is well documented that lower plasma ADIPOQ levels were associated with obesity [34]–[38], T2DM [39]–[43], dyslipidemia [44] and higher blood pressure [45]–[48]. Weight reduction has been shown to significantly increase plasma *ADIPOQ* levels [49]. Higher levels of ADIPOQ in plasma minimize the risk of developing T2DM [50]–[51]. Treatment with PPARγ2 agonist for hyperglycemia in T2DM patients [52]–[53] and treatment of hypertension with angiotensin-converting enzyme inhibitor or angiotensin II receptor antagonist [54]–[55] drastically increased the plasma *ADIPOQ* concentration.

**Figure 1:**
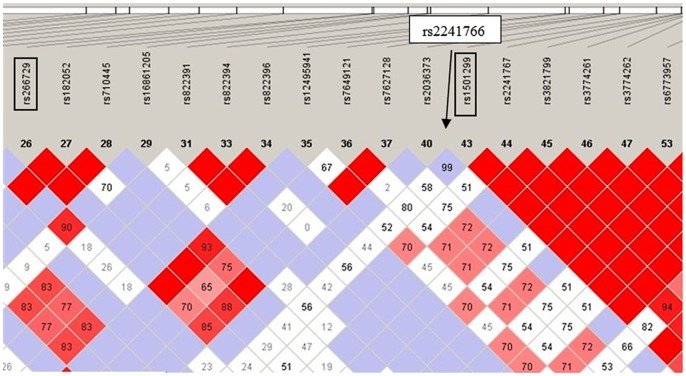
Linkage disequilibrium pattern of *ADIPOQ* SNPs in the Chinese population.

Linkage disequilibrium (LD) pattern of *ADIPOQ* SNPs in the Chinese population was shown in Figure 1. The SNPs rs266729 (−11377C>G in promoter), rs2241766 (+45T>G in exon 2) and rs1501299 (+276G>T in intron 2) in the *ADIPOQ* gene have been reported to be associated with MetS in Chinese populations [56]–[69]. However, these results have often been inconsistent due to a small sample size, which may affect their reliability.

In the present study, we aimed to investigate the association between *ADIPOQ* rs2241766, rs1501299, rs266729 polymorphisms and MetS in a Hubei Han Chinese population, and to systematically review the association of *ADIPOQ* rs2241766/rs266729/rs1501299 with MetS in Chinese via a meta-analysis.

## Materials and Methods

### Subjects

All participants gave written informed consent. The protocol was in accordance with the Helsinki Declaration, and was approved by the Institutional Review Boards of the Yiling Hospital in Yichang, Hubei Province. The present study included a total of 483 individuals of Hubei Han Chinese, comprising 322 MetS patients (93 males, 299 females, age 52.2±10.41 years) and 161 controls (84 males, 77 females, age 65.48±10.80 years). The IDF definition of MetS [5], which incorporates ethnicity by providing different criteria for the MetS in different ethnic groups, was used. IDF criteria for Asian identifies MetS for subjects with central obesity (waist circumference ≥90 cm for men, ≥80 cm for women), plus any two of: 1) elevated plasma TG≥1.69 mmol/L ; 2) low plasma HDL cholesterol <1.04 mmol/L for men, <1.29 mmol/L for women; 3) Systolic blood pressure (SBP)≥130 or diastolic blood pressure (DBP)≥85 mmHg or current medication; 4) fasting plasma glucose (FPG) ≥5.6 mmol/L or diagnosed with T2DM. Control subjects did not meet any IDF criteria of MetS.

### Clinical characteristics

The weight, height, waist and hip circumference were measured in all individuals. BMI and waist to hip ratio (WHR) were separately calculated as weight (kg)/height^2^ (m^2^) and waist (cm)/hip (cm). Clinical parameters measured included FPG, 2 hours' postprandial blood glucose (PBG), SBP and DBP, total cholesterol, TG, HDL-cholesterol, LDL-cholesterol, and fasting insulin.

### Genotyping

Genomic DNA was obtained from whole blood leukocytes using standard phenol/chloroform method. Three SNPs (rs2241766, rs1501299, rs266729) in the *ADIPOQ* gene were selected to genotype using polymerase chain reaction – restriction fragment length polymorphism (PCR-RFLP) method. The primer sequences and the annealing temperature of the PCR were shown in Table 1. PCR was performed in a total volume of 25 μl, containing 100 ng DNA template, 0.5 μl forward primer (20 μM), 0.5 μl reverse primer (20 μM), 0.5 μl Taq polymerase (2 U/μl), 2.5 μl 10×PCR buffer (Mg^2+^ Plus), 0.5 μl dNTP mixture. The PCR amplification conditions were as follows: an initial denaturing cycle at 94°C for 5 min, followed by 35 amplification cycles (denaturing at 94°C for 30 s, annealing for 30 s and extension at 72°C for 30 s), and a final extension at 72°C for 5 min.

**Table 1:** Primer sequences and reaction conditions for genotyping assay.

SNPs	Location	Primer sequences	Annealing temperature (°C)	Restriction enzyme	Fragment length (bp)
rs2241766	extron 2	F:5′-CAGCTCCTAGAAGTAGACTCTGCTG-3	61	SmaI	372, 209,163
		R:5′-GCAGGTCTGTGATGAAAGAGGCC-3′ [88]			
rs266729	promoter	F:5′-GGTGGACTTGACTTTACTGG-3′	60	HhaI	334,212,122
		R:5′-TAGAAGCAGCCTGGAGAA-3′ [89]			
rs1501299	intron2	F:5′-ATCAAGGTGGGCTGCAATA-3′	55	BsmI	654,452,202
		R:5′-TGGGAATAGGGATGAGGGT-3′ [89]			

### Association analysis

The genotype distribution in the cases and controls were separately tested for Hardy – Weinberg equilibrium using the χ ^2^ test before association analysis. The genotypic and allelic frequencies between MetS patients and controls were compared using χ ^2^ test. The genotype – disease association analyses were performed by logistic regression analysis. A *P* value less than 0.05 was considered statistically significant. Statistical analyses were performed using SPSS for Windows software (version 11.5). Statistical power was estimated by the ‘Case – control for discrete traits’ module of the web-based Genetic Power Calculator (http://pngu.mgh.harvard.edu/~purcell/gpc/qcc.html), which takes the control-case ratio of the study sample into account.

### Literature search strategy in meta-analysis

**Figure 2:**
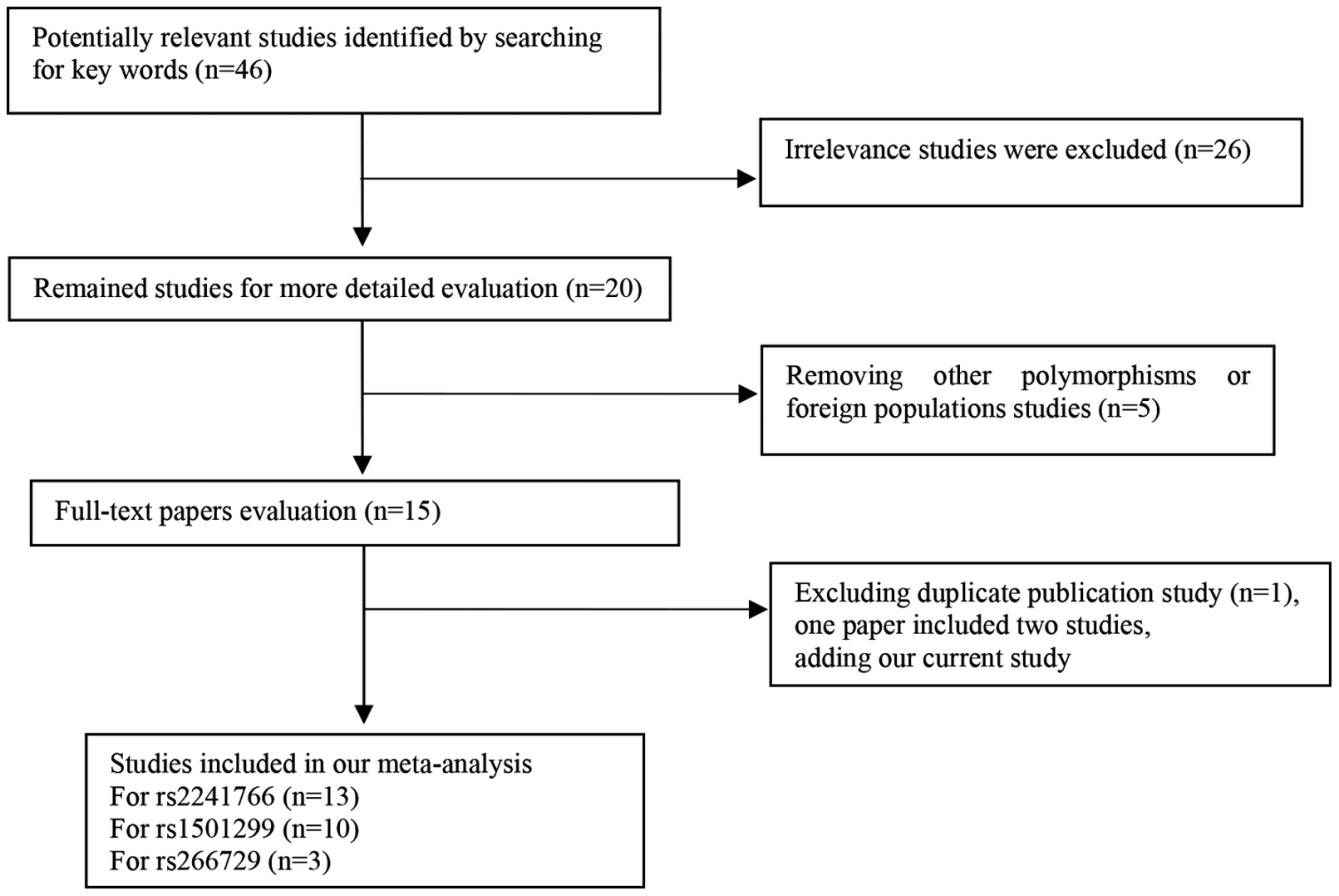
Flow diagram of studies included our meta-analysis.

PubMed and HuGeNet and China National Knowledge Infrastructure (CNKI) and VIP Information were searched up to August 2012, using “adiponectin” or “*ADIPOQ*” or “adiponectin gene polymorphism”, “AMP1”, and “metabolic syndrome” or “MetS” or “metabolic syndrome X” or “ syndrome X” as key words. The references of all computer-identified publications were searched for additional studies. The PubMed option “Related Articles” was used to search for potentially relevant papers. Reference lists in retrieved articles were also screened. Without any language restriction, we only selected published manuscripts (including their online supporting materials). Studies included in the meta-analysis must meet all the following criteria: (1) assessed the associations of polymorphisms in the *ADIPOQ* gene with MetS; (2) used case – control or cohort design; (3) provided odds ratio (OR) with 95 % confidence interval (CI), or genotype frequency among case and control group; (4) for duplicate publications from the same patient population, only the paper that had the largest population, contained more useful information or the latest one was selected. The following information was extracted: first author name, year of publication, ethnicity, sample size, genotype distribution and minor allele frequency in cases and controls, *p* value for allele frequency (Figure 2).

### Meta-analysis

We found forty-six published potentially relevant papers but only fourteen meet selection criteria. Among the forty-six papers, twenty-six were excluded due to irrelevance. In addition, four articles were excluded because they studied other polymorphisms or duplicate publications. Two studies from Korea and Croatia were also excluded. Moreover, one paper included two studies. From the above, 13 Chinese studies with a total of 2,684 cases and 2,864 controls were included in rs2241766 analysis, 11 Chinese studies with a total of 2,889 cases and 3,304 controls were included in rs1501299 analysis, 3 Chinese studies including our independent study with a total of 1,486 cases and 1,379 controls were included in rs266729 analysis. Table 2 summarized the characteristics of fifteen Chinese association studies of *ADIPOQ* polymorphisms with MetS.

**Table 2:** Characteristics of case-control studies included in a meta-analysis.

Study	Population	Group	Diagnostic Criteria	Sex (M/F)	Age	Subject Size	SNP rs266729 CC/CG/GG	G Allele Frequency (%)	*P*	SNP rs 2241766 TT/ GT/GG	G Allele Frequency (%)	*P*	SNP rs 1501299 GG/ TG/TT	G Allele Frequency (%)	*P*
Liu et al(2006) [56]	Chinese	case	IDF	86/70	53±11	156	–	–	–	73/66/17	32.1	0.315	–	–	–
		control		37/96	52±12	133	–	–	–	69/53/11	28.2		–	–	–
Yang et al(2007) [57]	Taiwan	case	IDF	–	–	658	–	–	–	–	–	–	295/318/45	69.0	0.117
		control		–	–	737	–	–	–	–	–	–	383/291/63	52.0	
Huang et al(2008) [58]	Shandong	case	CDS	164/60	77.4±2.9	224	–	–	–	118/92/14	26.8	0.796	131/83/10	77.0	**<0.001**
		control		149/51	77.9±2.8	200	–	–	–	108/80/12	26.0		103/75/22	70.3	
Li et al (2009) [59]	Chinese Han	case	IDF	54/34	50.1 ± 12.4	88	–	–	–	38/41/9	33.5	0.703	46/35/7	72.1	0.766
		control		25/31	33.4 ± 13.4	56	–	–	–	23/26/7	35.7		24/31/1	70.5	
Li et al(2010) [63]	Chinese Han	case	IDF	70/67	51.1±11.7	137	78/48/11	25.6	0.326	–	–	–	–	–	–
		control		55/76	35.7±13.8	131	63/60/8	29.3		–	–	–	–	–	–
Cai et al (2010) [60]	Chinese Han	case	IDF	–	–	38	–	–	–	15/18/5	37.0	0.212	20/16/2	73.7	0.218
		control		–	–	50	–	–	–	26/20/4	28.0		21/23/6	73.7	
Zhu et al (2010) [61]	Chinese Han	case	CDS	109/74	57.6±12.4	183	–	–	–	72/91/20	35.8	**0.018**	87/85/11	70.8	0.577
		control		90/54	56.6 ± 9.7	144	–	–	–	74/62/8	27.1		66/66/12	68.8	
Yao et al(2010) [62]	Beijing	case	WHO	65/124	45.7±8.0	189	–	–	–	91/79/18	30.6	0.57	92/90/7	72.5	**0.008**
		control		65/124	45.6±8.0	189	–	–	–	87/77/22	32.5		70/100/19	63.5	
Bu et al(2011) [64]	Jiangsu	case	CDS	116/79	61.3±12.4	195	–	–	–	76/97/22	36.2	**0.02**	93/89/13	70.5	0.646
		control		97/59	60.4±9.8	156	–	–	–	79/67/10	27.9		72/71/13	68.9	
Leu et al (2011) [65]	Taiwan	case	ATPIII	190/167	49.5±8.5	357	–	–	–	170/156/31	30.5	0.347	210/124/23	76.2	**0.022**
		control		305/300	43.5±4.0	605	–	–	–	307/251/47	28.5		313/238/54	71.4	
		case		218/312	–	530	–	–	–	264/224/42	29.1	0.866	335/174/21	79.6	**0.007**
		control		488/425	–	913	–	–	–	446/398/69	29.4		496/364/53	83.3	
Du et al(2011) [66]	Chinese Han	case	IDF	509/540	55.9±11.0	992	555/353/84	26.26	0.194	–	–	–	–	–	–
		control		491/601	55.7±13.1	1092	530/410/82	28.08		–	–	–	–	–	–
Wang et al(2012) [68]	Chinese Han	case	WHO	–	–	180	–	–	–	90/74/16	29.0	0.142	–	–	–
		control		25/25	43±1.9	50	–	–	–	31/16/3	22.0		–	–	–
Chen et al(2012) [67]	Ningxia	case	CDS	83/24	49.6±7.2	107	–	–	–	53/49/5	27.6	0.551	57/43/7	73.4	0.226
		control		66/36	48.7±5.6	102	–	–	–	59/35/8	25.0		61/38/3	80.4	
Li et al (2012) [69]	Sichuan	case	CDS	–	51.6±11.6	116	–	–	–	71/40/5	21.6	0.19	–	–	–
		control		–	51.8±11.5	108	–	–	–	76/28/4	16.7		–	–	–
Gao et al(2012)	Hubei	case	IDF	93/229	52.2±10.4	322	188/103/9	20.20	0.117	147/158/17	29.8	**0.033**	157/139/24	70.8	0.068
		control		84/77	66.4±10.3	161	85/65/6	24.70		93/61/7	23.3		64/82/15	65.0	

The associations of polymorphisms in the *ADIPOQ* gene with MetS were estimated by calculating pooled OR and 95% CI under additive, dominant and recessive genetic models by using stata 10.0 software. ORs were calculated using 2×2 contingency tables for each study. The χ ^2^-based Q-test and the inconsistency index (*I^2^*) were applied to assess heterogeneity among studies. Sensitivity analysis was conducted by removing one study at a time and calculating the pooled ORs for the remaining studies. The Z-test was used to calculate the *P* value of the overall effect for the meta-analysis. Pooled ORs were computed by the fixed-effects method of Mantel-Haenszel (peto method) for data combined under no heterogeneity between studies (*P*>0.1). If significant heterogeneity exists between studies (*P*≤0.1), then a random effects model of Der-Simonian-Laird (D-L method) is appropriate for data combined. The conservative Egger's regression analysis was used to evaluate publication bias.

## Results

### Clinical characteristics of subjects

Clinical characteristics of the study subjects are shown in Table 3. Independent *t*-test analysis showed that the gender, age, height, weight, waist circumference, hip circumference, SBP, DBP, FPG, BMI, triacylglycerol, HDL-cholesterol, and LDL-cholesterol, in MetS were significantly higher than those of the controls group. Since height, weight, waist circumference, hip circumference, SBP, DBP, FPG, BMI, triacylglycerol, HDL-cholesterol, and LDL-cholesterol were components of MetS, only gender and age were used as covariates to be adjusted in association analyses.

**Table 3:** Clinical characteristics of the study subjects.

Characteristics	Case group	Control group	*P* value
gender (M/F)	93/229	84/77	<0.001
Age (years)	52.2±10.41	65.48±10.80	<0.001
Height (cm)	155.93±7.99	153.44±7.80	0.001
Weight (kg)	71.89±11.83	50.72±12.32	<0.001
Waist circumference (cm)	91.74±6.55	69.86±7.89	<0.001
Hip circumference (cm)	101.38±5.79	86.57±6.02	<0.001
SBP (mmHg)	145.60±24.45	115.63±11.37	<0.001
DBP (mmHg)	91.92±13.13	72.14±8.05	<0.001
FPG (mmol/L)	6.87±3.01	4.98±0.42	<0.001
BMI (kg/m^2^)	28.90±2.71	21.55±3.87	<0.001
Total cholesterol	4.91±1.81	1.81±1.06	0.916
Triacylglycerol	3.16±2.85	1.01±0.32	<0.001
HDL-cholesterol	1.46±0.48	1.76±0.43	<0.001
LDL-cholesterol	2.81±3.19	2.06±0.74	0.003

SBP: systolic blood pressure DBP: diastolic blood pressure FPG: fasting plasma glucose TC: total cholesterol TG: triglyceride HDL: high-density lipoprotein LDL: low density lipoprotein.

### SNP rs2241766 and MetS

The genotypic distributions of the rs2241766 polymorphism was in Hardy-Weinberg equilibrium in both MetS patients and controls group (*P*>0.05). The G allele and GG+TG genotype frequencies of rs2241766 in MetS patients were significantly higher than those of controls group (G allele: 29.8% vs 23.3%, OR = 1.40, 95%CI = 1.03–1.91, *P* = 0.033; GG+TG genotype: *P* = 0.012, OR = 1.63, 95%CI = 1.11–2.39). The logistic regression analysis adjusted by gender and age showed a nominally significant association for rs2241766 GG+GT genotype (*P* = 0.065, OR = 1.55, 95%CI = 0.97–2.48).

**Figure 3:**
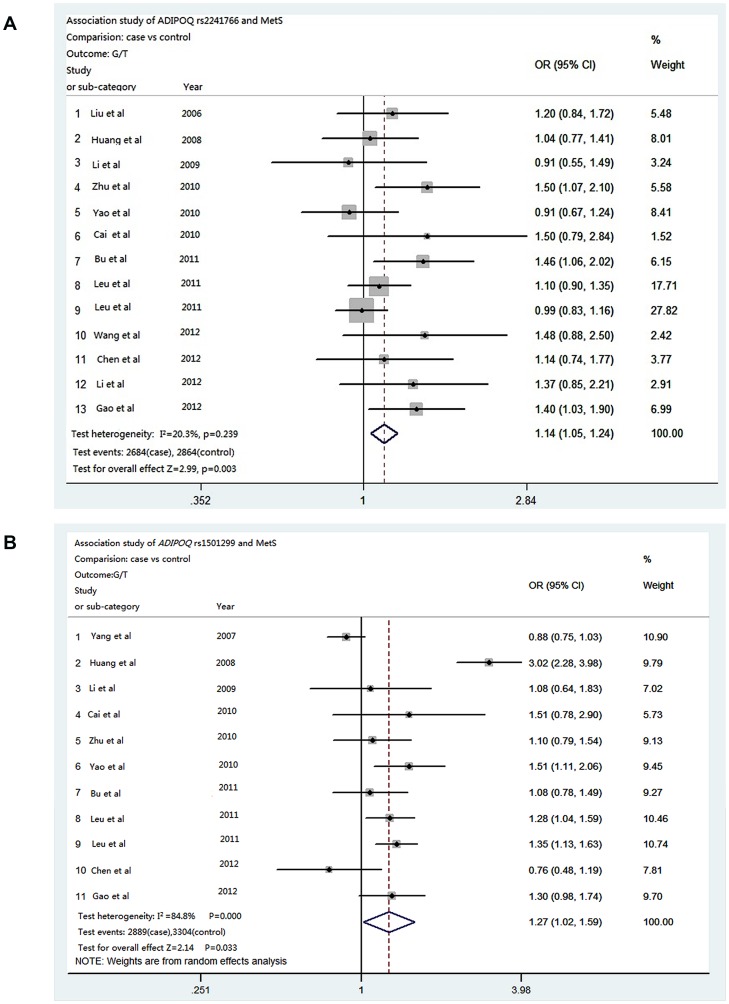
Forest plots of meta-analysis of the association of *ADIPOQ* rs2241766 (A) and rs1501299 (B) polymorphisms with metabolic syndrome in the Chinese population. Estimation of odds ratios (OR) and 95% confidence intervals (CI) in each study are displayed as closed square and horizontal line, respectively. The size of the black squares reflects the weight of the study in the meta-analysis. The diamond represents the combined OR, calculated using a random or fixed effect model, with its 95%CI.

We identified thirteen Chinese studies for the *ADIPOQ* rs2241766 including our study. Figure 3(A) presents the forest plot of risk G allele OR of individual study and meta-analysis for association between *ADIPOQ* rs2241766 and MetS in a total of 2,684 case patients and 2,864 control subjects. Of these, eleven studies showed a trend of elevated OR for the risk allele G. Two studies have an opposite trend. There was no significant between study heterogeneity. A fixed effect model was thus used and generated a combined allelic OR of 1.14 (95%CI 1.04–1.24, *P* = 0.003) for the rs2241766 G allele, 1.19 (95%CI 1.07–1.33, *P* = 0.002) for the GG+GT genotypes in dominant model (Table 4). We further combined genotype data of all thirteen studies. The SNP rs2241766 showed consistent associations with MetS: OR = 1.12 (95%CI = 1.04–1.22, *P* = 0.005) for the G allele, OR = 1.17 for GG+GT genotypes (95%CI = 1.06–1.30, *P* = 0.003) in dominate model.

**Table 4:** Meta-analysis of associations between polymorphisms in the *ADIPOQ* gene and MetS risk.

Model	OR(95%CI)	*P* value	*P* for heterogeneity (I^2^%)	*P* for publication bias
**rs2241766**				
GG vs TT additive model	1.23(1.00–1.52)	0.051	0.586 (0.0)	0.289
GG vs TG additive model	1.06(0.86–1.31)	0.586	0.952 (0.0)	0.681
GG+TG vs TT dominant model	1.19(1.07–1.33)	**0.002**	0.231 (21.0)	0.044
GG vs TG+TT recessive model	1.15(0.94–1.41)	0.180	0.831 (0.0)	0.564
G vs T allele	1.14(1.04–1.24)	**0.003**	0.239 (20.3)	0.074
**rs1501299**				
GG vs TT additive model	1.48(1.20–1.82)	**<0.001**	0.132 (33.4)	0.937
GG vs TG additive model	1.16(0.95–1.41)	0.147	**0.002** (64.7)	0.431
GG+TG vs TT dominant model	1.42(1.16–1.75)	**0.001**	0.297 (15.4)	0.612
GG vs TG+TT recessive model	1.20(0.99–1.46)	0.062	**0.001** (65.6)	0.490
G vs T allele	1.27(1.02–1.59)	**0.033**	**0.000** (84.8)	0.654

*P* values <0.05 were shown in bolded types.

### SNP rs266729 and MetS

No association was observed for either rs266729 G allele, GG genotype in recessive model or GG+CG genotypes in dominant model with or without adjustment for gender and age in our sample. Because only three association studies of the rs266729 polymorphism with MetS were found in Chinese, we pooled genotype data of all three studies. The allele frequencies for G were 0.249 in the MetS group, and 0.278 in the control group. The pooled OR was 0.862 (95%CI = 0.76–0.97, *P* = 0.015) for the G allele, 0.80 (95%CI = 0.68–0.92, *P* = 0.003) for GG+GT genotypes in dominant model.

### SNP rs1501299 and MetS

An association trend was observed for rs1501299 G allele (OR = 1.30, 95%CI 0.98–1.74, *P* = 0.068) and the GG genotype in recessive model without (OR = 1.48, 95%CI 1.01–2.18, *P* = 0.045) or with adjustment of gender and age (OR = 1.54, 95%CI 0.97–2.45, *P* = 0.066) in our sample.

Figure 3 (B) presents the forest plot of risk G allele OR of individual study and meta-analysis for association between *ADIPOQ* rs1501299 and MetS in a total of 2,889 cases and 3,304 controls1 from eleven Chinese studies. Nine studies showed a trend of elevated OR for the risk allele G. One study from the Taiwan [57] and one study from Ningxia [67] showed a trend in the opposite direction. Significant associations were found for the G allele (OR = 1.27, 95%CI 1.02–1.59, *P* = 0.033), the GG+TG genotypes in dominant model (OR = 1.42, 95%CI 1.16–1.75, *P* = 0.001) and for GG vs TT in additive model (OR = 1.48, 95%CI 1.20–1.82, *P*<0.001) (Table 4).

### Sensitivity analysis

A sensitivity analysis was conducted by removing one study at a time and calculating the pooled ORs for the remaining studies. The results indicated that none of the individual studies influenced the pooled ORs (1.12–1.20, 95%CI 1.02–1.32), *P* (0.014–0.001) value and heterogeneity (*I* ^2^ = 2.3∼26.9%) for rs2241766. However, the result of meta-analysis for rs1501299 was not stable. The meta-analysis result of the allele model becomes significant after removing Yang's [57] (*P* = 0.009, OR = 1.33, 95%CI = 1.08–1.66) or Chen's [67] (*P* = 0.014, OR = 1.33, 95%CI = 1.06–1.67) studies that showed an association trend in the opposite direction.

### Heterogeneity analysis

A significant heterogeneity was observed for rs1501299 in allele analysis (*P<*0.001, *I* ^2^ = 84.8%). Meta-regression analysis including covariates the age in case groups and control groups, publication date, sample size, diagnostic criteria, region and gender, showed that only the age in case groups and control groups significantly contributed to the heterogeneity (*P* = 0.008, 0.031, respectively). The inconsistency index *I* ^2^ decreases from 84.8% to 60.3% after removing Huang's study [58] that had the highest age in case groups and control groups.

## Discussion

In the present study we examined the association of rs2241766, rs1501299 and rs266729 polymorphisms in the *ADIPOQ* gene with MetS risk in Chinese. The rs2241766 was associated with susceptibility to MetS in the Hubei Han Chinese population (allele G: 29.8% vs 23.3%, *P* = 0.033). The association was further confirmed by our meta-analysis, which involved 2,684 case patients and 2,864 control in Chinese populations. A weak association was found for the rs1501299 GG genotype in recessive model in our sample, and significant associations were found for the G allele (OR = 1.27, 95%CI 1.02–1.59, *P* = 0.033), the GG+TG genotypes in dominant model (OR = 1.42, 95%CI 1.16–1.75, *P* = 0.001) and for GG vs TT in additive model (OR = 1.48, 95%CI 1.20–1.82, *P*<0.001) in meta-analysis. The result from pooled three genotype data suggested that rs266729 was significantly associated with MetS in additive (G allele: OR = 0.862, 95%CI = 0.76–0.97, *P* = 0.015) and dominant models (GG+GT genotypes: OR = 0.80, 95%CI = 0.68–0.92, *P* = 0.003). Therefore, both our study in Hubei Han Chinese and meta-analyses in the Chinese population suggested that polymorphisms in the *ADIPOQ* gene were associated with MetS risk.

To our knowledge, this study represents the first meta-analysis between polymorphisms in the *ADIPOQ* gene and MetS in the Chinese populations. Thirteen small studies previously conducted in Chinese populations examined the rs2241766 polymorphism in relation to MetS with inconsistent results. Only two studies [61], [64] found significant associations, which are similar to our results. Our meta-analysis consistently supports the association between the SNP rs2241766 and MetS under additive, dominant models, and in the pooled data. For rs1501299, a weak association was found in our sample, and significant associations were detected in meta-analysis. For rs266729, although no association was observed in our Hubei Han Chinese and two previous studies [63], [66], a significant association was detected for pooled genotype data. The G allele may decrease the risk of MetS (OR = 0.862, 95%CI = 0.76–0.97, *P* = 0.015). These results highlight the pivotal role of systematic review to draw firm conclusions.

The low serum ADIPOQ level is a strong risk factor for MetS [36], [38], [70]–[71]. It is well documented that ADIPOQ levels are highly heritable (30–70%) [72]–[74]. Candidate gene study, GWAS and meta-analysis have shown pronounced associations between common polymorphisms in the *ADIPOQ* gene and plasma ADIPOQ levels [74]–[78]. Vasseur et al. [73] demonstrated that higher ADIPOQ levels were associated with variant alleles of SNPs rs2241766 T>G (*P* = 0.01) and rs1501299 G>T (*P* = 0.01), whereas variant alleles at SNP rs266729 C>G (*P* = 0.0003) were associated with a lower ADIPOQ level. Patients with rs266729CG, CG+GG genotypes (*P* = 0.034, 0.035 respectively) had higher levels of serum ADIPOQ than those with the CC genotypes in a Chinese Han population [66]. Another study found that the GG genotype for rs1501299 was associated with lower serum ADIPOQ levels as compared with the GT and TT genotypes [79]. Moreover, a meta-analysis by Menzaghi et al. [74] indicated that variants in *ADIPOQ* played a role in modulating ADIPOQ secretion. Therefore, polymorphisms in the *ADIPOQ* gene may regulate the serum ADIPOQ levels, thereby influence the risk of MetS.

Six meta-analysis of the association between the genetic variants in the *ADIPOQ* gene and T2DM have been published [74], [80]–[84]. Menzaghi et al. [74] found no association between any of the SNPs (rs2241766/rs1501299/rs266729) and T2DM among populations from all over the world. Li et al. [83] and Chen et al. [84] demonstrated that the rs2241766 G allele increased the risk of T2DM (OR = 1.34, 1.28, respectively) in Chinese populations. However, no association was detected in the meta-analysis of 6370 T2DM patients and 15443 normal individuals from all over the world [81]. In contrast, Gong et al. [81] and Han et al. [82] consistently demonstrated that the G allele of rs266729 contributed to the development of T2DM in global meta-analysis of populations from all over the world and European White, but not Asian. For the rs1501299 polymorphism in intron 2, although associations were found in meta-analysis of Li et al's [80] nine and Chen et al's [84] eight case – control studies in the Chinese Han population, there was no association in meta-analysis of Li et al's [83] eleven Chinese Han case – control studies and Han et al's [82] global meta-analysis of populations from all over the world. Recently, two meta-analyses explored the relationship between SNPs rs2241766 and rs1501299 in the *ADIPOQ* gene and blood pressure and essential hypertension in Chinese populations [85]–[86]. No significant association was found. *ADIPOQ* rs1501299 T (OR = 1.59; 95% CI 1.39–1.81) was associated with an increased risk of obesity in a recent meta-analysis [87]. These results implicated *ADIPOQ* as a pleiotropic locus for MetS and its components, presumably serving as an important physiological and pharmacological target in the prevention and treatment of MetS.

Although we limit our meta-analysis to the Chinese population, meta-analysis still revealed significant between-study heterogeneity for SNP rs1501299 (Table 3). The source of between-study heterogeneity may be due to: 1) Regional variations. All of the subjects in the current meta-analysis were Han Chinese but from different regions and the Han Chinese population is not a genetically homogenous group. 2) Different diagnostic criteria for MetS. IDF [56]–[57], [59]–[60], [63], [66], CDS [58], [61], [64], [67], [69], WHO [62], [68] and ATPIII [65] diagnostic criteria are used respectively. 3) Selection bias. The age of the control subjects ranges from 33.4 [59] to 77.9 [58], and sample size ranges from 88 [60] to 2141 [66]. Our meta-regression analysis showed that the age in case groups and control groups significantly contributed to the heterogeneity.

We acknowledged that there were some limitations in our study. First, sample size in our study was comparatively small and had insufficient statistical power to detect the association. Assuming the prevalence of 30%, and the minor allele frequencies of the marker and QTL are both 0.2, a marker is in complete LD (D' = 1) with a QTL, and using a dominant genetic model, this study had about 68% power at a significance level of α = 0.05 to detect an effect size of 1.4. Second, the present meta-analysis was based primarily on unadjusted effect estimates and the confounding factors were not controlled for. Third, due to lack of original data, the effects of gene – gene and gene-environment interactions were not considered in our current study. In addition, a weak publication bias was detected for the rs2241766 dominant model.

In conclusion, our results and meta-analysis demonstrates that *ADIPOQ* is a pleiotropic locus for MetS and its components in Chinese Han population. In the future, well-designed studies with large sample sizes in diverse ethnic populations are warranted.
